# Visualizing the dynamic mechanical power and time burden of mechanical ventilation patients: an analysis of the MIMIC-IV database

**DOI:** 10.1186/s40560-023-00709-9

**Published:** 2023-11-29

**Authors:** Han Chen, Zhi-Zhong Chen, Shu-Rong Gong, Rong-Guo Yu

**Affiliations:** 1 The Third Department of Critical Care Medicine, Shengli Clinical Medical College of Fujian Medical University, Fujian Provincial Hospital, Fujian Provincial Center for Critical Care Medicine, Fujian Provincial Key Laboratory of Critical Care Medicine, Dongjie 134, Gulou District, Fuzhou, Fujian China; 2 General Product Center, Fujian Foxit Software Development, Joint Stock Co. Ltd., Building 5, Area G, Fuzhou Software Park, No. 89 Software Avenue, Gulou District, Fuzhou, Fujian China

**Keywords:** Mechanical power, Mechanical ventilation, Respiratory failure, MIMIC-IV, Mortality

## Abstract

**Background:**

Limiting driving pressure and mechanical power is associated with reduced mortality risk in both patients with and without acute respiratory distress syndrome. However, it is still poorly understood how the intensity of mechanical ventilation and its corresponding duration impact the risk of mortality.

**Methods:**

Critically ill patients who received mechanical ventilation were identified from the Medical Information Mart for Intensive Care (MIMIC)-IV database. A visualization method was developed by calculating the odds ratio of survival for all combinations of ventilation duration and intensity to assess the relationship between the intensity and duration of mechanical ventilation and the mortality risk.

**Results:**

A total of 6251 patients were included. The color-coded plot demonstrates the intuitive concept that episodes of higher dynamic mechanical power can only be tolerated for shorter durations. The three fitting contour lines represent 0%, 10%, and 20% increments in the mortality risk, respectively, and exhibit an exponential pattern: higher dynamic mechanical power is associated with an increased mortality risk with shorter exposure durations.

**Conclusions:**

Cumulative exposure to higher intensities and/or longer duration of mechanical ventilation is associated with worse outcomes. Considering both the intensity and duration of mechanical ventilation may help evaluate patient outcomes and guide adjustments in mechanical ventilation to minimize harmful exposure.

**Supplementary Information:**

The online version contains supplementary material available at 10.1186/s40560-023-00709-9.

## Background

Energy must be applied to the respiratory system in order to expand the lungs in patients receiving positive-pressure mechanical ventilation (MV). The concept of mechanical power has been introduced to describe the amount of energy applied to the lungs per unit of time during MV [1]. This measure combines various ventilatory variables, such as tidal volume (VT), driving pressure, and respiratory rate (RR), to estimate the intensity of energy delivered to the lungs. Lung-protective ventilation strategies aim to reduce the mechanical energy and power applied to the lungs during MV, consisting of lower end-inspiratory (plateau) airway pressures, lower VT, and the application of positive end-expiratory pressures (PEEP). Clinical studies have demonstrated that these strategies can improve outcomes [2–4].

Recent studies have shown that limiting driving pressure and mechanical power is associated with reduced mortality risk in both patients with and without acute respiratory distress syndrome [5–10]. However, most studies considered only the baseline mechanical power (e.g., within the first 24 h of MV) as the risk factor. Indeed, mechanical power, as the measurement of the intensity of MV per unit of time, must be considered together with the factor of time regarding the potentially harmful effects and patient outcomes. In a large, registry-based, prospective cohort study, the impact of time-varying exposure to varying intensities of MV (measured by dynamic driving pressure or mechanical power) on ICU mortality was investigated in patients with acute respiratory failure [5]. The study revealed that even short periods of cumulative exposure to higher intensities of MV were harmful. However, ventilation parameters were collected at 08:00 a.m. each day in that study, thus limiting the availability of more precise information regarding the relationship between different cumulative exposure times and clinical outcomes. In our study, we aim to utilize data from an electronic database with more detailed records to visualize the interaction between the duration and intensity of MV.

## Methods

### Database

The data for this study were obtained from the Medical Information Mart for Intensive Care-IV (MIMIC-IV) database [11], which contains de-identified data from patients who were admitted to critical care units at the Beth Israel Deaconess Medical Center. Consent was obtained for the original data collection at the time of the establishment of the database; therefore, the Institutional Review Board of Fujian Provincial Hospital waived the need for informed consent for this study. The study protocol was approved by the Institutional Review Board of Fujian Provincial Hospital on September 01, 2023 (approval number 2023-MQSP-0901-01) and was conducted between September 01 and October 15, 2023. The study was designed and conducted in accordance with relevant guidelines and regulations (Declaration of Helsinki).

### Data extraction and study population

Data were extracted by Dr. Han Chen and Dr. Shu-Rong Gong (database access certification number: HC 36014736, SRG 35606844). PostgreSQL tools Ver. 10.16 were used for data extraction as previously reported [12–14]. The following data were extracted by using Structured Query Language (SQL): age, gender, weight, co-morbidities, duration of MV, survival time, length of hospital stay, length of ICU stay, sequential organ failure assessment (SOFA) score, simplified acute physiology score-II (SAPS-II), and blood gas analysis results. Besides, the time and value of MV parameters, including peak inspiratory pressure (Ppeak), PEEP, measured RR, and VT, were also extracted for further calculation. We did not extract plateau pressure because the data for plateau pressure were noticeably fewer compared to the data for other MV parameters.

All patients who were invasively ventilated were screened for inclusion. The inclusion criteria were: (1) Baseline clinical characteristic data available; (2) MV data available; (3) Age ≥ 18 years. The exclusion criteria were: (1) The second episode of MV during the ICU stay; (2) The second admission to ICU; (3) MV duration < 72 h; (4) MV data after the 28th days of MV were excluded from the analysis.

### Missing data and calculations

Missing data on MV parameters were imputed by using the values from the nearest available time point or, alternatively, by using the median if no nearby value was available. The highest and lowest extreme values were replaced by the 99% and the 1% percentiles (by the “winsor2” command in STATA), respectively. The missing data on MV parameters accounted for less than 1% of the total data (Additional file 1: Table S1).

The dynamic driving pressure was calculated as Ppeak minus PEEP. Dynamic mechanical power was calculated as 0.098 × RR × VT × (Ppeak – 0.5 × dynamic driving pressure) [5]. MV data at all time points were collected from the database (typically every 4 h, which may vary depending on the individual condition), thus allowing us to calculate the corresponding dynamic mechanical power and its associated exposure duration (until the next recorded time point). Multiplying the mechanical power by the exposure duration gives the mechanical energy, which represents the total ‘dose’ of MV for that time period. By summing the mechanical energy throughout the entire MV period, the total mechanical energy delivered can be calculated. Dividing the total mechanical energy by the total duration of MV gives the average mechanical power level for the entire MV period.

### Visualization method

**Fig. 1:**
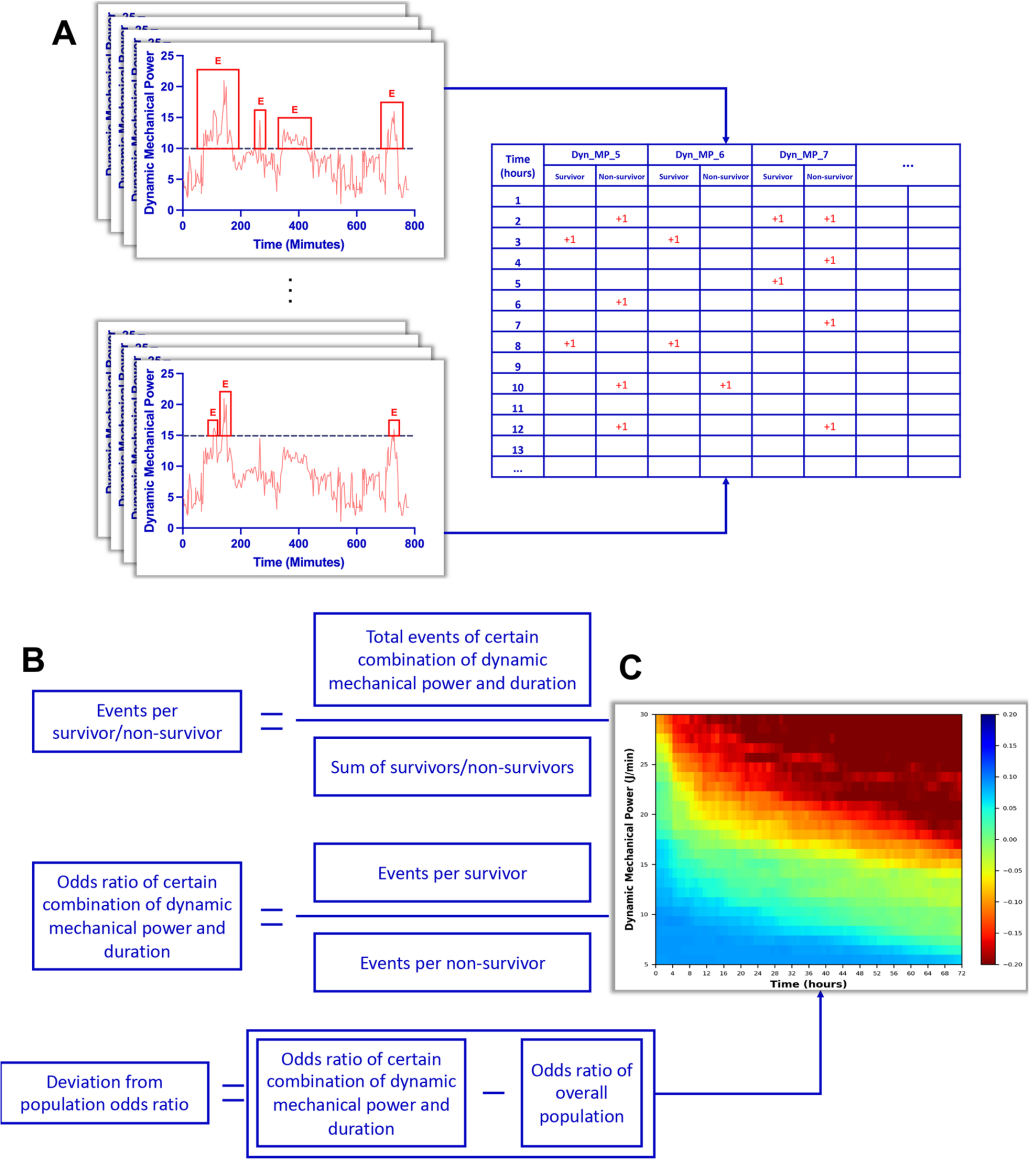
Diagram of the data visualization method. **A** The dynamic mechanical power was calculated for each continuous time interval. An event (E) was defined when the dynamic mechanical power exceeded a specific threshold (e.g., 10 in the upper left and 15 in the lower left), as indicated by the red boxes. Kindly note that the two examples illustrate the same variation of dynamic mechanical power over the same period of time (for illustrative purposes only, not the entire mechanical ventilation process). However, the calculated number of events was different due to the use of different thresholds. This counting process was performed for each patient at every combination of time (ranging from 1 to 72 h) and dynamic mechanical power (ranging from 5 to 30 J/min) levels. The counts were recorded in the sheet displayed on the right. **B** The average event count per survivor/non-survivor, odds ratio, and deviation of odds ratio from the overall population were calculated. Please refer to the text for a detailed explanation. **C** The relationship between different time-intensity combinations and mortality was visualized using a heatmap, illustrating the deviations in odds ratio

We used a modified visualization method adapted from the one used to assess the impact of duration and intensity of intracranial pressure insults on the 6-month neurological outcome [15]. The visualization method, as illustrated in Fig. 1, was to examine the association between MV intensity and 28-day mortality. As previously described, we calculated the dynamic mechanical power for each continuous time interval, considering it as an event if the dynamic mechanical power exceeded a certain threshold and its duration was recorded (Fig. 1A). Event counts were computed for each patient at different combinations of time (ranging from 1 to 72 h) and dynamic mechanical power (ranging from 5 to 30 J/min) levels. For example, when considering a threshold of 10 J/min, any instance where the mechanical power exceeds 10 J/min was recorded as one event, along with its corresponding duration of exposure. Subsequently, different combinations of intensity and duration were considered, and the number of exposures was calculated. Taking an exposure of 10 J/min for 4 h as an example, any 10 J/min event with an exposure exceeding 4 h would contribute one additional record for the combination of 10 J/min and 4 h. The average event count per survivor/non-survivor at a specific time-intensity combination was calculated by dividing the total event count at this combination by the number of survivors/non-survivors. The ratio of average event counts per patient between survivors and non-survivors was calculated for each time-intensity combination, which gives the odds ratio (OR). By subtracting the overall OR (the ratio between total event counts for survivors and non-survivors), we were able to determine the OR deviation of each time-intensity combination from the overall OR (Fig. 1B). A positive deviation suggests a favorable effect on survival, while a negative deviation suggests an unfavorable effect. OR deviations were visualized in a heatmap to illustrate the relationship between different time-intensity combinations and mortality (Fig. 1C). Additionally, the percentage of the deviation from the overall OR was also calculated and presented in a heatmap. Based on the distribution of OR values, we fitted curves using the percentile option method for OR deviations of − 0.1 and − 0.2 to indicate time-intensity combinations that result in a 10% and 20% increase in risk, respectively. We also plotted the fitting curves on a grid for better visualization and to determine the corresponding mortality risk for different combinations of MV duration and intensity.

### Statistical analysis

Continuous variables were presented as median with interquartile ranges (IQR), and the Wilcoxon rank-sum test was used. Categorical variables were presented as counts (percentages) and compared using the chi-square test. Patients were grouped based on quartiles of average dynamic mechanical power for Kaplan–Meier survival analysis and Log-Rank test to evaluate its impact on survival. Univariate and multivariate COX proportional hazards model analysis with stepwise elimination was conducted to assess the factors associated with mortality risk. Variables with a *p*-value < 0.2 were considered for inclusion in the multivariate analysis. STATA (ver. 15.1, StataCorp., TX, USA) was used for data manipulation and analysis. The heatmap was plotted by using Python (Ver. 3.8) with Matplotlib (Ver. 3.7.0), which is a widely used data visualization library [16]. All reported *p-*values were two-sided, and a *p* < 0.05 was considered significant. This article is reported following the STROBE guidelines [17].

## Results

**Fig. 2:**
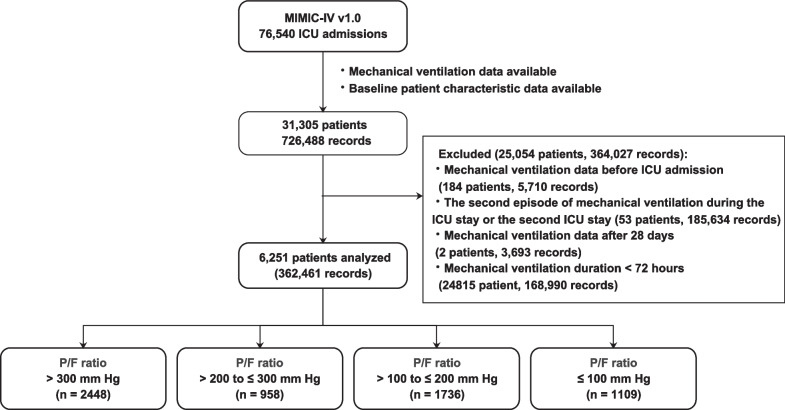
Flowchart showing a step-by-step selection of patients included in the study. *ICU* intensive care unit, *MIMIC* Medical Information Mart for Intensive Care

A total of 6251 patients were included, and the 28-day mortality was 29.7% (4394 survivors and 1857 non-survivors, Fig. 2). Baseline patient characteristics are summarized in Table 1. In brief, the patients had a median (IQR) age of 65 (53, 75) years, and 3612 (57.8%) of them were men. There were 3803 (60.8%) patients with hypoxemic respiratory failure. Among these patients, 958 (15.3%) had an arterial oxygen partial pressure to the fraction of inspired oxygen ratio (*P*/*F* ratio) of > 200 to ≤ 300 mmHg, 1736 (27.8%) had a *P*/*F* ratio of > 100 to ≤ 200 mmHg, and 1109 (17.7%) had a *P*/*F* ratio of ≤ 100 mmHg. Patients with lower *P*/*F* ratios had higher SOFA and SAPS-II scores.

**Table 1:** Baseline clinical characteristics of patients

	All patients (n = 6251)	Stratification by first day *P*/*F* ratio			
		> 300 mm Hg (n = 2448)	> 200 to ≤ 300 mm Hg (n = 958)	> 100 to ≤ 200 mm Hg (n = 1736)	≤ 100 mm Hg (n = 1109)
Age (years)	65 (53, 75)	65 (54, 76)	66 (55, 77)	65 (53, 75)	62 (52, 73)
Male	3612 (57.8%)	1341 (54.8%)	547 (57.1%)	1049 (60.4%)	675 (60.9%)
Weight (kg)	80.6 (67.7, 98.1)	76 (64.6, 91.5)	81.1 (67.9, 98)	85.7 (70.2, 103.3)	86 (70.3, 102)
Height (cm)	170 (165, 175)	170 (163, 173)	170 (163, 175)	170 (165, 175)	170 (165, 175)
SOFA score	7 (4, 10)	4 (2, 6)	5 (3, 8)	9 (6, 11)	10 (8, 13)
SAPS-II score	44 (34, 55)	37 (29, 47)	44 (35, 55)	48 (39, 59)	52 (42, 63)
Co-morbidities					
Congestive heart failure	2021 (32.3%)	727 (29.7%)	324 (33.8%)	567 (32.7%)	403 (36.3%)
Cerebrovascular disease	1260 (20.2%)	657 (26.8%)	199 (20.8%)	262 (15.1%)	142 (12.8%)
Chronic pulmonary disease	1796 (28.7%)	643 (26.3%)	275 (28.7%)	534 (30.8%)	344 (31%)
Diabetes without complication	1595 (25.5%)	592 (24.2%)	226 (23.6%)	472 (27.2%)	305 (27.5%)
Diabetes with complication	614 (9.8%)	247 (10.1%)	97 (10.1%)	156 (9%)	114 (10.3%)
Malignant cancer	749 (12%)	316 (12.9%)	100 (10.4%)	195 (11.2%)	138 (12.4%)
Renal disease	1442 (23.1%)	556 (22.7%)	226 (23.6%)	387 (22.3%)	273 (24.6%)
Severe liver disease	580 (9.3%)	214 (8.7%)	83 (8.7%)	173 (10%)	110 (9.9%)

Data are presented as median (interquartile range) for continuous variables and counts (percentages) for categorical variables

*P/F ratio* partial pressure of oxygen to fraction of inspired oxygen ratio, *SAPS-II* simplified acute physiology score-II, *SOFA* sequential organ failure assessment

We calculated the average values of MV parameters throughout the entire MV process, regardless of the duration of the exposure. The median *P*/*F* ratio was 162 (101, 257) mmHg, Ppeak was 19.7 (16.2, 23.6) cm H_2_O, PEEP was 6.1 (5, 8.4) cm H_2_O, RR was 20.7 (18, 23.5) min^−1^, VT was 460 (404, 518) mL and was 7.0 (6.3, 7.9) mL/kg per predicted body weight. Patients with a lower *P*/*F* ratio had higher Ppeak, PEEP, RR, and lower VT. The total dynamic mechanical ventilation energy was 105214 (65091, 184169) J, while the average dynamic mechanical power was 11.8 (9, 15.8) J/min when the total dynamic mechanical ventilation energy was divided by the MV duration. An increase in both total dynamic mechanical ventilation energy and the average dynamic mechanical power was observed as the *P*/*F* ratio decreased (Table 2).

**Table 2:** Mechanical ventilation data and outcomes

	All patients (n = 6251)	Stratification by first day *P*/*F* ratio			
		> 300 mm Hg (n = 2448)	> 200 to ≤ 300 mm Hg (n = 958)	> 100 to ≤ 200 mm Hg (n = 1736)	≤ 100 mm Hg (n = 1109)
*P*/*F* ratio (mmHg)	162 (101, 257)	375 (333, 433)	244 (220, 270)	143 (120, 168)	73 (59, 86)
Peak inspiratory pressure (cm H_2_O)	19.7 (16.2, 23.6)	17.7 (14.7, 21.6)	18.7 (15.9, 22.2)	20.6 (17.6, 24.3)	23.1 (19.7, 26.7)
Positive end-expiratory pressure (cm H_2_O)	6.1 (5, 8.4)	5 (4.9, 6.7)	5.5 (4.9, 7)	7 (5.4, 8.9)	8.8 (6.9, 11.2)
Measured respiratory rate (min^−1^)	20.7 (18, 23.5)	19.9 (17.2, 22.5)	19.9 (17.6, 22.5)	21.1 (18.4, 23.9)	22.8 (19.8, 25.7)
Tidal volume (mL)	460 (404, 518)	455 (399, 512)	461 (405, 521)	468 (410, 523)	457 (400, 521)
Tidal volume per predicted body weight (mL/kg)	7.0 (6.3, 7.9)	7.0 (6.3, 7.9)	7.1 (6.3, 8.0)	7.1 (6.3, 7.9)	6.9 (6.2, 7.7)
Total dynamic mechanical ventilation intensity (J)	105,214 (65,091, 184,169)	84,269 (55,780, 142,501)	89,756 (60,396, 151,851)	116,859 (74,919, 196,094)	162,460 (96,696, 274,257)
Averaged dynamic mechanical power (J/min)	11.8 (9, 15.8)	10 (8, 13)	10.8 (8.8, 13.7)	13.1 (10.1, 16.9)	15.9 (12.1, 20.6)
SaO_2_ (%)	97 (95, 98)	98 (97, 99)	97 (96, 98)	96 (94, 98)	96 (92, 98)
SpO_2_ (%)	98 (95, 100)	100 (97, 100)	99 (97, 100)	97 (95, 99)	96 (93, 99)
PaO_2_ (mmHg)	106 (80, 149)	151 (103, 200)	119 (101, 156)	95 (77, 124)	83 (63, 107)
PaCO_2_ (mmHg)	39 (34, 45)	37 (32, 43)	39 (34, 44)	39 (34, 45)	41 (36, 50)
Lactate (mmol/L)	1.7 (1.2, 2.8)	1.5 (1.1, 2.3)	1.6 (1.1, 2.5)	1.8 (1.2, 2.9)	2.1 (1.4, 3.6)
Outcomes					
ICU mortality at 28 days	1857 (29.7%)	717 (29.3%)	261 (27.2%)	499 (28.7%)	380 (34.3%)
ICU mortality	1588 (25.4%)	567 (23.2%)	230 (24%)	446 (25.7%)	345 (31.1%)
Duration of mechanical ventilation (days)	6 (4.2, 9.5)	5.7 (4, 8.9)	5.7 (3.9, 9)	6.1 (4.2, 9.5)	7.1 (4.7, 11.1)
Length of ICU stay (days)	9.9 (6.7, 15.4)	9.9 (6.7, 15.4)	9.4 (6.3, 14.9)	9.7 (6.7, 15)	10.9 (7.1, 16.6)

Data are presented as median (interquartile range) for continuous variables and counts (percentages) for categorical variables

*P/F ratio* partial pressure of oxygen to fraction of inspired oxygen ratio, *PaO*_*2*_ arterial partial pressure of oxygen, *PaCO*_*2*_ arterial partial pressure of carbon dioxide, *SaO*_*2*_ peripheral arterial oxygen saturation, *SpO*_*2*_ pulse oxygen saturation, *ICU* intensive care unit

**Fig. 3:**
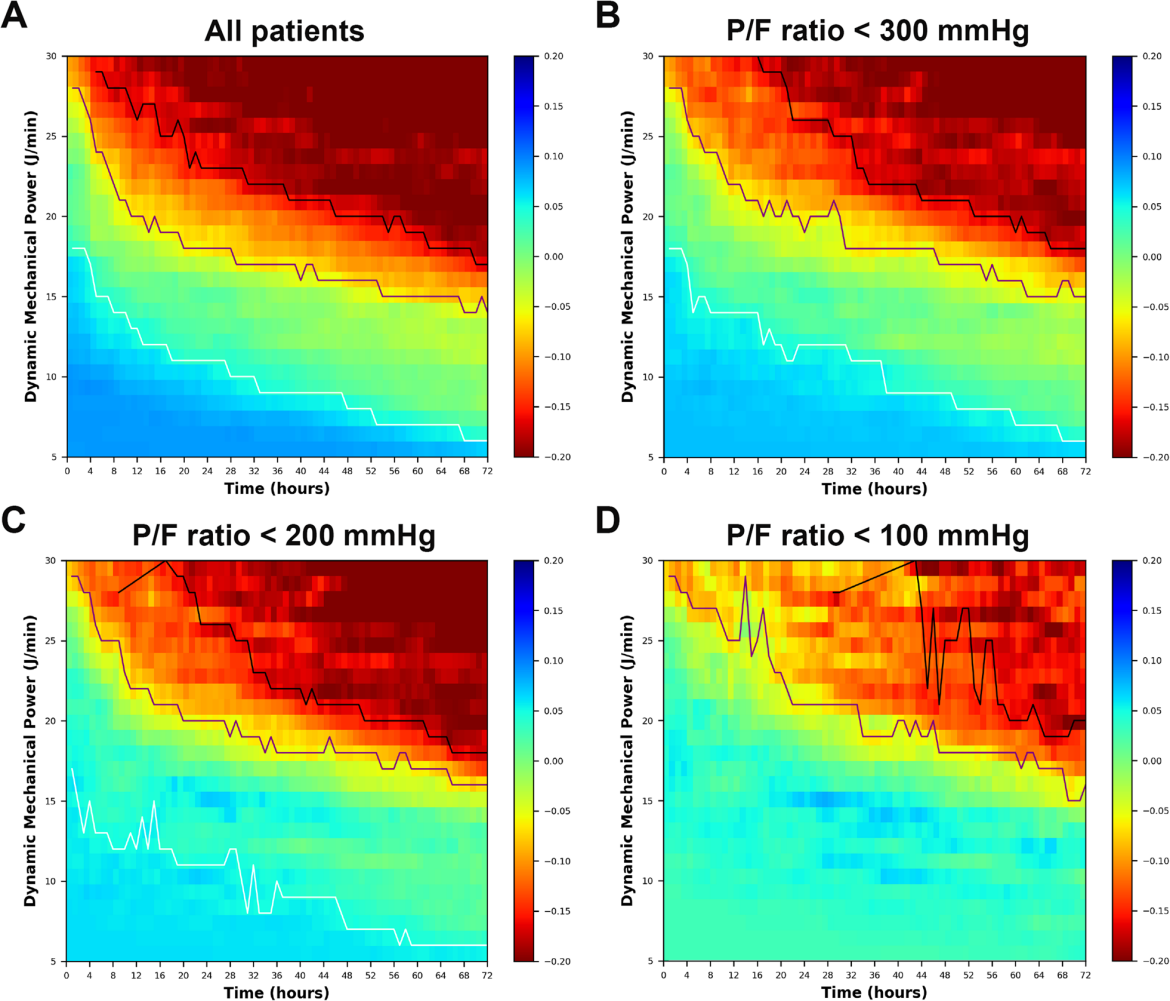
Heatmap illustrating the absolute odds ratio deviation. A positive deviation indicating a favorable effect was represented in blue, while a negative deviation suggesting an unfavorable effect was represented in red. The white fitting curve represents the odds ratio deviation close to zero (the “transition curve”), indicating the transition into the region of insult types. The purple line and the black line represent the odds ratio deviation close to − 0.1 and − 0.2, respectively, indicating a 10% and 20% increase in mortality risk

The color-coded plots in Fig. 3 visualize the correlations between the 28-day mortality and the different combinations of MV intensity and the duration of exposures. Two distinct regions can be observed: a predominantly blue zone in the lower left region, indicating the combination of lower dynamic mechanical power and shorter duration of exposure can occur more frequently in patients with better survival, while a predominantly red zone was observed in the upper right region, indicating the combination of higher dynamic mechanical power and longer duration of exposure can occur more frequently in patients with worse outcome. A similar trend was observed among the overall population and patients with respiratory failure with different severity (*P*/*F* ratio < 300, 200, or 100 mmHg, respectively). The fitting curves follow an approximately exponential pattern: for higher MV intensities, the transition occurs at shorter exposure durations and verse visa. When percentage changes of OR were used instead of the absolute differences, similar trends can be observed (Additional file 1: Fig. S1).

**Fig. 4:**
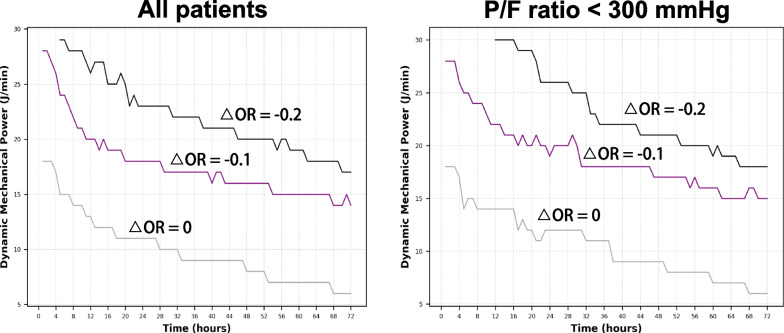
Grid plots of fitting curves for mortality risk. The heatmap has been modified to emphasize the distinct fitting curves. The purple curve represents a 10% increase in the time-intensity combination for mortality risk, while the black curve represents a 20% increase in risk. To enhance visibility against the white background, the curve representing a mortality risk of 0% (previously white) has been adjusted to gray

By plotting the fitting curves on grids, we were able to identify the potential deleterious MV intensities within any specific timeframes. In the overall population (Fig. 4A), ventilation at a dynamic mechanical power of 18 J/min for 24 h was associated with a 10% increase in mortality risk, while a dynamic power of 23 J/min for 24 h was associated with a 20% increase in mortality risk. Similarly, when ventilated for 48 h, a 10% increase in mortality risk corresponded to a dynamic mechanical power of 16 J/min, while a 20% increase in mortality risk corresponded to 20 J/min. When MV was sustained for 72 h, an increase in mortality risk of 10% and 20% corresponded to dynamic powers of 14 J/min and 16 J/min, respectively. When considering the patients with respiratory failure (i.e., P/F ratio < 300 mmHg, Fig. 4B), 24-h MV at intensities of 19 J/min and 26 J/min was associated with a 10% and 20% increase in mortality risk, respectively. Similarly, a 48-h MV intensity of 17 J/min and 21 J/min was associated with a 10% and 20% increase in mortality risk, respectively. Ventilation at intensities of 15 J/min and 18 J/min for 72 h was associated with a 10% and 20% increase in mortality risk, respectively. Similar trends can be observed when percentage changes of OR were used instead of the absolute differences (Additional file 1: Fig. S2).

**Fig. 5:**
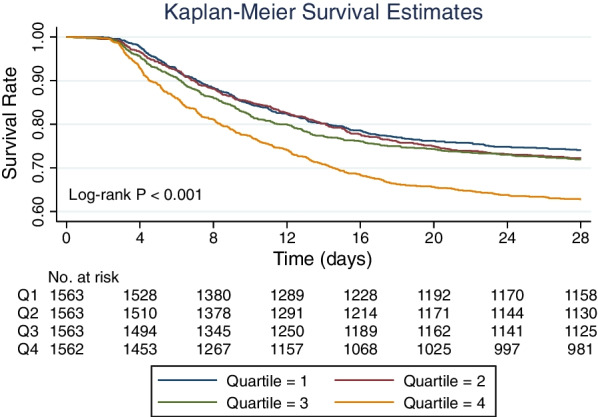
Kaplan–Meier survival analysis of patients with varying average dynamic mechanical power. The patients were divided into four groups based on the quartiles of their average dynamic mechanical power. It was observed that patients with higher average dynamic mechanical power (quartile 4, with an average dynamic mechanical power of > 15.8 J/min) had a significantly lower probability of survival (*p* < 0.001)

Patients were stratified for survival analysis according to the quartiles of the average dynamic mechanical power, which indicates the averaged intensity of the entire MV period. It was observed that patients in quartile 4 (i.e., average dynamic mechanical power > 15.8 J/min) had a significantly higher mortality rate (*p* < 0.001, Fig. 5). In the COX regression model, dynamic mechanical power was identified as a risk factor for mortality (hazard ratio 1.06, 95% confidence interval 1.05–1.07, Table 3). Congestive heart failure, diabetes, and renal disease were identified as potential risk factors in the univariate analysis (i.e., *p* < 0.2) but were excluded from the final model in the stepwise selection process. The model encompassing these variables is presented as a result of sensitivity analysis in the Additional file 1: Table S2. Dynamic mechanical power was still identified as a risk factor for mortality in the sensitivity analysis.

**Table 3:** Multivariate analysis of the risk factors of mortality

	Univariate analysis			Multivariate analysis		
	Hazard ratio	95% CI	*p* value	Hazard ratio	95% CI	*p* value
Age	1.02	1.02–1.02	< 0.001	1.02	1.02–1.03	< 0.001
Gender	1.13	1.03–1.24	0.009	1.31	1.19–1.44	< 0.001
SOFA score	1.07	1.06–1.08	< 0.001	1.02	1–1.03	0.080
SAPS-II score	1.02	1.02–1.03	< 0.001	1.01	1.01–1.02	< 0.001
Averaged dynamic mechanical power	1.04	1.03–1.05	< 0.001	1.06	1.05–1.07	< 0.001
Congestive heart failure	1.17	1.06–1.28	0.001			
Cerebrovascular disease	1.44	1.29–1.59	< 0.001	1.68	1.51–1.87	< 0.001
Chronic pulmonary disease	1.04	0.94–1.15	0.442			
Diabetes	1.11	0.95–1.28	0.181			
Renal disease	1.33	1.2–1.47	< 0.001			
Malignant cancer	1.46	1.29–1.65	< 0.001	1.31	1.15–1.49	< 0.001
Severe liver disease	1.63	1.43–1.86	< 0.001	1.91	1.65–2.21	< 0.001

*CI* confidence interval, *SAPS-II* simplified acute physiology score-II, *SOFA* sequential organ failure assessment

## Discussion

The main findings of the present study were: (1) both the intensity and duration of dynamic mechanical power exposure were associated with the risk of mortality and can be visualized; (2) even low levels of mechanical power exposure may increase the risk of death as the duration of mechanical ventilation increases.

A key factor in improving outcomes for patients receiving MV is preventing secondary lung injury, known as ventilator-induced lung injury. Ventilator-induced lung injury occurs due to the interplay between ventilator settings and the condition of the lung tissue. In addition to managing the underlying lung condition, extensive research has been conducted on various MV parameters, such as pressures [18], volume [19], flow [20], and respiratory rate [21], to promote lung protection. In recent years, a new composite index that integrates these factors, known as mechanical power, has received increasing attention. Considering mechanical power as a whole may provide better insights than examining its individual components separately. However, there is currently a lack of research on the relationship between mechanical power and its duration of exposure and mortality. A recent study utilized daily data to investigate the duration of exposure to mechanical power and found a correlation between longer exposure and increased risk of mortality [5]. However, estimating the daily situation based on a single time point may introduce bias. Additionally, there is a need for a more intuitive approach to help researchers and clinicians assess the relationship between exposure duration, intensity, and mortality risk. Therefore, in this study, we developed a visual method to demonstrate the relationship between any time-intensity combination and the mortality risk.

By calculating the ORs of survival for each combination of MV duration and intensity, we can easily determine the risk associated with each combination, which is a straightforward approach. Comparing the OR with the overall population OR (by subtraction), we can calculate the relative risk of any combination. This allows us to create a curve representing different risk levels, similar to contour lines in geography, and guide the selection of safe thresholds for limiting mechanical power in different time frames. For instance, we can intuitively indicate that for patients undergoing MV for 48 h, a dynamic mechanical power exceeding 16 J/min is linked to a 10% increase in mortality risk. Taking this combination as an example for counting, we found that this exposure was observed in 1525 out of the 4394 survivors (34.7%) and in 834 out of the 1857 non-survivors (44.9%), indicating a combination of moderate mechanical power and exposure duration that poses a potential risk is widely present among patients undergoing mechanical ventilation. It is worth noting that our analysis considered both the overall population and a subset of patients with a *P/F* ratio < 300 mmHg, which indicates respiratory failure. This decision was based on two factors. First, a similar trend was observed in the subgroup with *P*/*F* ratios < 200 mmHg. Second, the sample size for the subgroup with *P*/*F* ratios < 100 mmHg was too small, leading to significant fluctuations in the fitting curve and making it impossible to fit a curve representing a risk of 0%. Still, we demonstrated the results in the Additional file 1: Figs. S2, S3).

We included not just patients with acute respiratory distress syndrome but all patients who received MV in the ICU, which ensures the generalizability of the study. Our data suggests that even relatively low levels of dynamic mechanical power (approximately 15 J/min) over a relatively long duration (72 h) were associated with an increased mortality risk, consistent with previous research findings [5]. Indeed, it is unlikely to establish a causal relationship through a retrospective study. However, our findings indicate that cumulative exposure to higher intensities and/or longer duration of MV was associated with worse outcomes. Therefore, clinicians should consider evaluating the potential necessity and feasibility of reducing mechanical power and the potential clinical benefits it may offer.

It is worth noting that, similar to previous studies [5, 7, 22], static measurements of airway pressure (i.e., plateau pressure data) were unavailable in the majority of patients, as clinicians often only pay more attention to changes in respiratory mechanics in more severe respiratory failure patients. Urner et al. reported a dramatically higher plateau pressure than peak airway pressure in the previous study [5], which seems counterintuitive since plateau pressure is generally expected to be lower than or equal to peak pressure, as the latter is determined by both airway resistance and respiratory system compliance while the former is only determined by compliance. However, this can be attributed to the fact that only a tiny proportion of patients in their study had plateau pressure measurements (1633 out of 13,408 patients). Although no explicit data supports this, it is reasonable to speculate that these patients had more severe conditions and were, therefore, more likely to have had static respiratory mechanics measured. For this reason, we chose not to calculate static compliance and driving pressure based on plateau pressure but instead used peak airway pressure to calculate dynamic driving pressure and dynamic mechanical power. Another interesting difference is that in our study, the average dynamic driving pressure in the most severe subgroup (i.e., *P*/*F* ratio < 100 mmHg) was 15.9 J/min, which was obviously lower than the 19 J/min reported in previous studies [5]. This may reflect the heterogeneity among the populations included, as evident from the markedly different composition ratios; our study's subgroup with *P/F* ratio < 100 mmHg accounted for 1109 out of 6251 (17.76%), while theirs was 767 out of 13,408 (5.72%). Nevertheless, our study, as well as the previous study, found a significant association between elevated dynamic mechanical power and mortality in different populations, underscoring the importance of monitoring it.

Lung injury is primarily caused by transpulmonary driving pressure, which refers to the pressure exerted on the alveoli. However, it is essential to note that airway pressure, although it changes in parallel with transpulmonary pressure, can also be influenced by additional factors such as spontaneous breathing and chest wall compliance. As a result, airway pressure may not always exhibit the same changes as transpulmonary pressure, making it insufficient to rely solely on airway pressure to reflect the lungs' condition accurately. This is one of the limitations of this study. Unfortunately, transpulmonary pressure has not been widely used in clinical practice, and therefore, we decided to analyze the data using airway pressure instead. This decision aims to ensure that the results of this study have better generalizability.

Our observations indicate that the mortality rate also increases as the duration of MV increases. This could potentially indicate that a longer MV duration leads to a poorer prognosis regardless of the specific mechanical power involved. Therefore, we divided patients into different exposure groups based on the quartiles of average mechanical power and found that higher average mechanical power was associated with mortality, indicating that high mechanical power is a risk factor for death. Moreover, we performed a multivariate analysis, which also suggests that higher mechanical power is associated with a higher mortality rate. The hazard ratio was 1.06, which is very close to the previous study by Urner et al*.* with a hazard ratio of 1.060 (95% credible interval 1.053–1.066) [5].

This study has several limitations that should be acknowledged. First, as mentioned earlier, we did not conduct calculations based on transpulmonary pressure. Second, it was a retrospective study based on electronic healthcare records, which is limited by the nature of the retrospective design and the data source used. Therefore, establishing causality is challenging, and further controlled trials are warranted. Third, while we calculated the ORs and expected them to represent relative risk, they are not completely equivalent. Future research should consider calculating relative risks to provide more accurate risk prediction tools using the visualization method. Fourth, our study only focused on a 72-h timeframe, excluding patients who did not receive MV for a full 72 h. This could introduce bias. In fact, we attempted to include all patients in the visualization analysis, regardless of their MV duration. We observed a deviation in the fitting curves after approximately 24 h (Additional file 1: Fig. S3), which may be due to the inclusion of a heterogeneous population with varying durations of MV. On the other hand, extending the time frame would further reduce the sample size.

## Conclusions

Cumulative exposure to higher intensities and/or longer duration of MV is associated with worse outcomes. Considering both the intensity and duration of MV may help evaluate patient outcomes and guide adjustments in MV to minimize harmful exposure.

### Supplementary Information

**Additional file 1: Table S1**. Description of missing data. **Table S2**. Multivariate model encompassed all potential confounders. **Figure S1**. Heatmap illustrating the percentage odds ratio deviation. **Figure S2**. Grid plots of fitting curves for mortality risk using percentage odds ratio deviation. **Figure S3**. Grid plots of fitting curves for mortality risk of the P/F ratio < 200 mmHg and the P/F ratio < 100 mmHg subgroups. **Figure S4**. Heatmap and fitting curve illustrating the odds ratio deviation in all mechanical ventilation patients.

## Data Availability

The data that support the findings of this study are available from the MIMIC-IV database, but restrictions apply to the availability of these data, which were used under license for the current research and so are not publicly available. Data are, however, available from the authors upon reasonable request and with permission of the holder of the database.

## Abbreviations

ICU: Intensive care unit
IQR: Interquartile ranges
MIMIC: Medical Information Mart for Intensive Care
MV: Mechanical ventilation
OR: Odds ratio
PEEP: Positive end-expiratory pressure
P/F ratio: Partial pressure of oxygen to fraction of inspired oxygen ratio
RR: Respiratory rate
SAPS: Simplified acute physiology score
SOFA: Sequential organ failure assessment
SQL: Structured Query Language
VT: Tidal volume
