# Supplementary material for: Visualizing the dynamic mechanical power and time burden of mechanical ventilation patients: an analysis of the MIMIC-IV database
Source: J Intensive Care. 2023 Nov 29;11:58. doi: 10.1186/s40560-023-00709-9 (PMC10685677; doi:10.1186/s40560-023-00709-9)

**Visualizing the dynamic mechanical power and time burden of mechanical ventilation patients: an analysis of the MIMIC-IV database**

Han CHEN, M.D. ^1^; Zhi-Zhong CHEN, E.B. ^2^; Shu-Rong GONG, M.D. ^1^; Rong-Guo YU, M.D. ^1, *^

**Author’s Affiliation:**

1. The Third Department of Critical Care Medicine, Shengli Clinical Medical College of Fujian Medical University, Fujian Provincial Hospital, Fujian Provincial Center for Critical Care Medicine, Fujian Provincial Key Laboratory of Critical Care Medicine, Fuzhou, Fujian, China

2. General Product Center, Fujian Foxit Software Development Joint Stock Co., Ltd, Fuzhou, Fujian, China

**Additional File**

**Materials**

This supplement has additional information on methods and results, organized as follows:

1. Table S1 Description of missing data.
2. Table S2 Multivariate model encompassed all potential confounders
3. Figure S1 Heatmap illustrating the percentage odds ratio deviation
4. Figure S2 Grid plots of fitting curves for mortality risk using percentage odds ratio deviation
5. Figure S3 Grid plots of fitting curves for mortality risk of the P/F ratio < 200 mm Hg and the P/F ratio < 100 mm Hg subgroups
6. Figure S4 Heatmap and fitting curve illustrating the odds ratio deviation in all mechanical ventilation patients

**Table S1 Description of missing data**

| **Variable** | **Total counts** | | **Number of missing data** | | **Percentage of missingness (%)** | |
| --- | --- | --- | --- | --- | --- | --- |
|  | **Patients** | **Records** | **Patients** | **Records** | **Patients** | **Records** |
| Tidal volume | 32,449 | 727,930 | 3,317 | 4,573 | 10.2 | 0.6 |
| Respiratory rate | 32,449 | 727,930 | 3,417 | 4,065 | 10.5 | 0.6 |
| Peak inspiratory pressure | 32,449 | 727,930 | 2,562 | 5,944 | 7.9 | 0.8 |
| PEEP | 32,449 | 727,930 | 3,308 | 4,401 | 10.2 | 0.6 |
| PaO_2_ | 6,251 | 362,461 | 1,217 | 63,089 | 19.5 | 17.4 |
| PaCO_2_ | 6,251 | 362,461 | 1,217 | 63,089 | 19.5 | 17.4 |
| P/F ratio | 6,251 | 362,461 | 1,657 | 87,140 | 26.5 | 24.0 |
| Age | 6,251 | 362,461 | 0 | 0 | 0 | 0 |
| Gender | 6,251 | 362,461 | 0 | 0 | 0 | 0 |
| Height | 6,251 | 362,461 | 1,450 | 77,281 | 23.2 | 21.3 |
| Weight | 6,251 | 362,461 | 29 | 1,127 | 0.5 | 0.3 |
| SOFA score | 6,251 | 362,461 | 0 | 0 | 0 | 0 |
| SAPS-II score | 6,251 | 362,461 | 0 | 0 | 0 | 0 |

**Table S2 Multivariate model encompassed all potential confounders**

|  | **Hazard ratio** | **95% CI** | **P value** |
| --- | --- | --- | --- |
| **Age** | 1.02 | (1.02, 1.03) | < 0.001 |
| **Gender** | 1.31 | (1.19, 1.45) | < 0.001 |
| **SOFA score** | 1.01 | (1.00, 1.03) | 0.110 |
| **SAPS-II score** | 1.01 | (1.01, 1.02) | < 0.001 |
| **Averaged dynamic mechanical power** | 1.06 | (1.05, 1.07) | < 0.001 |
| **Congestive heart failure** | 1.03 | (0.93, 1.14) | 0.523 |
| **Cerebrovascular disease** | 1.69 | (1.52, 1.88) | < 0.001 |
| **Diabetes** | 1.01 | (0.86, 1.18) | 0.946 |
| **Renal disease** | 1.06 | (0.95, 1.19) | 0.311 |
| **Malignant cancer** | 1.32 | (1.16, 1.50) | < 0.001 |
| **Severe liver disease** | 1.92 | (1.66, 2.22) | < 0.001 |

This model encompassed all potential confounders, including variables that were excluded from the final model in the stepwise selection process, as a result of sensitivity analysis.

CI = confidence interval, SAPS-II = simplified acute physiology score-II, SOFA = sequential organ failure assessment.

**Figure S1 Heatmap illustrating the percentage odds ratio deviation**

The difference between this figure and Figure 3 in the text lies in presenting the risk associated with different combinations of mechanical ventilation intensity and duration using the relative change (in percentage) of odds ratios. The calculation was dividing the obtained odds ratio deviation by the overall population's odds ratio to get the percentage shift.

The white fitting curve represents the percentage odds ratio deviation close to zero. The purple and black lines represent the percentage odds ratio deviation close to -0.1 and -0.2, respectively, indicating a 10% and 20% increase in mortality risk.

**Figure S2 Grid plots of fitting curves for mortality risk using percentage odds ratio deviation**

Figure S1 has been modified to emphasize the distinct fitting curves. The purple curve represents a 10% increase in the time-intensity combination for mortality risk, while the black curve represents a 20% increase in risk. Please note that due to the small sample size in the subgroup of P/F ratios < 100 mm Hg, significant fluctuations were observed in the fitting curve, and it was impossible to fit a curve representing a risk of 0%.


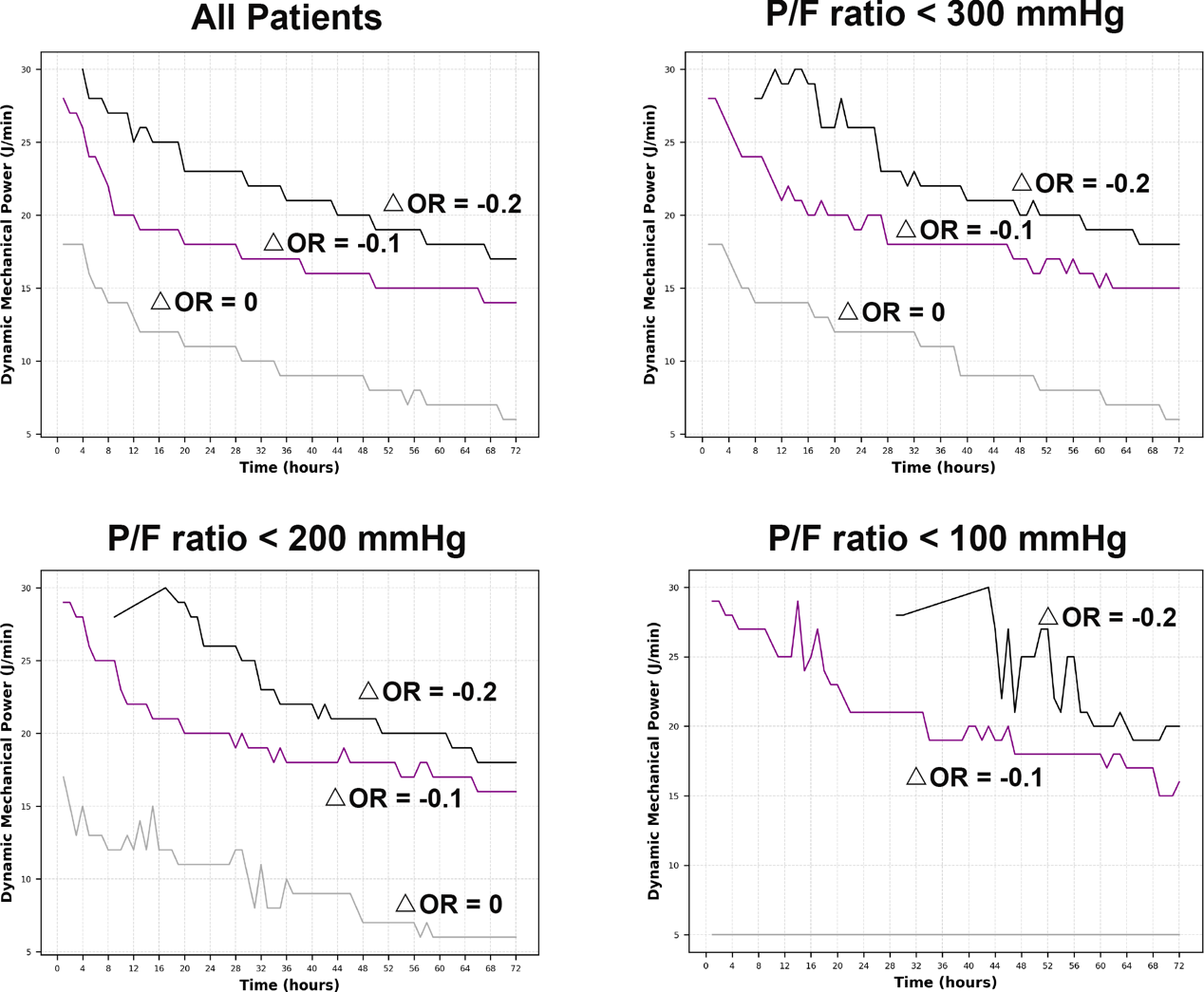


**Figure S3 Grid plots of fitting curves for mortality risk of the P/F ratio < 200 mm Hg and the P/F ratio < 100 mm Hg subgroups**

These two subgroups were not shown in Figure 4 for the following reasons: first, a similar trend was observed in the subgroup with P/F ratios < 200 mm Hg. Second, the sample size for the subgroup with P/F ratios < 100 mm Hg was too small, leading to significant fluctuations in the fitting curve and making it impossible to fit a curve representing a risk of 0%.


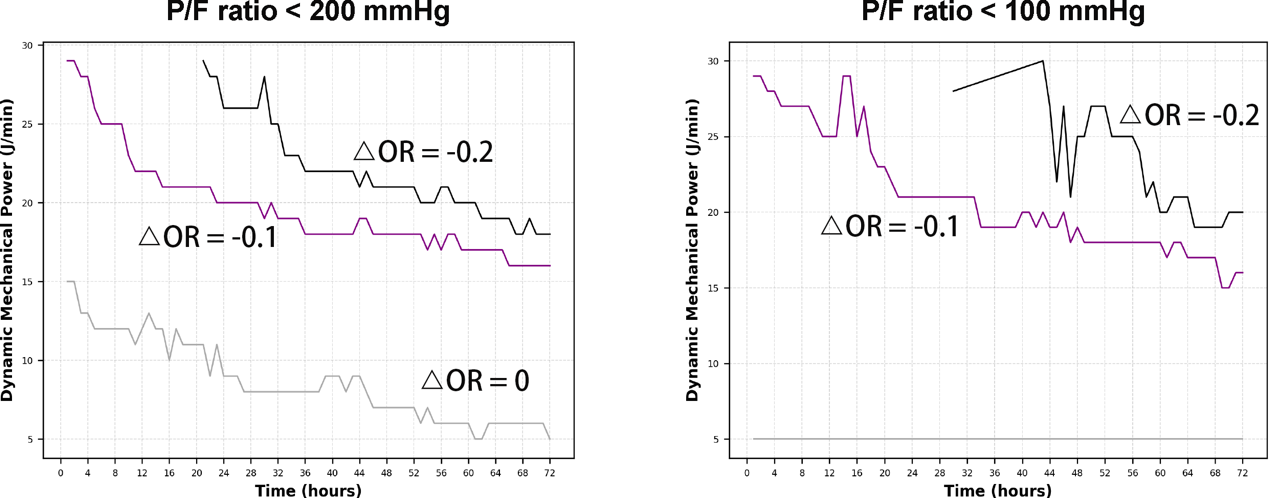


**Figure S4 Heatmap and fitting curve illustrating the odds ratio deviation in all mechanical ventilation patients**

Patients with a mechanical ventilation duration of < 72 hours were also involved in the analysis. A deviation in the fitting curves can be observed after approximately 24 hours, which may be due to the inclusion of a heterogeneous population with varying durations of mechanical ventilation.


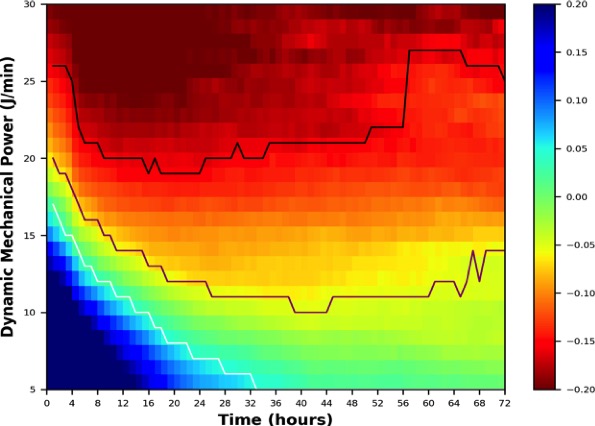


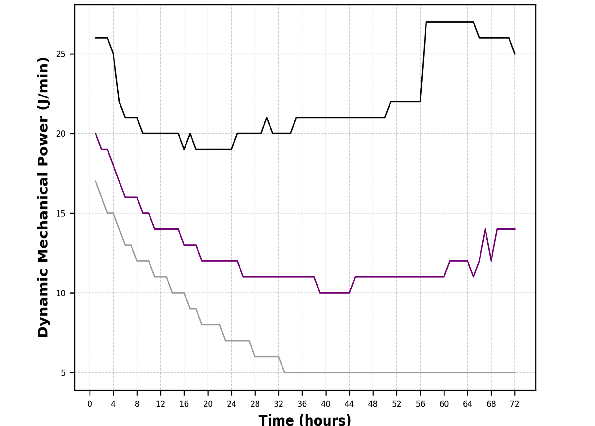

Supplement: Supplementary file 1 — Additional file 1: Table S1. Description of missing data. Table S2. Multivariate model encompassed all potential confounders. Figure S1. Heatmap illustrating the percentage odds ratio deviation. Figure S2. Grid plots of fitting curves for mortality risk using percentage odds ratio deviation. Figure S3. Grid plots of fitting curves for mortality risk of the P/F ratio < 200 mmHg and the P/F ratio < 100 mmHg subgroups. Figure S4. Heatmap and fitting curve illustrating the odds ratio deviation in all mechanical ventilation patients. [file 40560_2023_709_MOESM1_ESM.docx]
